# Requirement for Serine-384 in Caspase-2 processing and activity

**DOI:** 10.1038/s41419-020-03023-6

**Published:** 2020-10-03

**Authors:** Alexey V. Zamaraev, Pavel I. Volik, Dmitry K. Nilov, Maria V. Turkina, Aleksandra Yu. Egorshina, Anna S. Gorbunova, Svetlana Iu. Iarovenko, Boris Zhivotovsky, Gelina S. Kopeina

**Affiliations:** 1 Faculty of Medicine, MV Lomonosov Moscow State University, 119991 Moscow, Russia; 2 Belozersky Institute of Physicochemical Biology, MV Lomonosov Moscow State University, 119991 Moscow, Russia; 3 Faculty of Medicine and Heath Sciences, Department of Clinical and Experimental Medicine, Linköping University, 58185 Linköping, Sweden; 4 Division of Toxicology, Institute of Environmental Medicine, Karolinska Institute, Box 210, 17177 Stockholm, Sweden

**Keywords:** Proteases, Cell death

## Abstract

Caspase-2 is a unique and conservative cysteine protease which plays an important role in several cellular processes including apoptotic cell death. Although the molecular mechanisms of its activation remain largely unclear, a major role belongs to the architecture of the caspase-2 active center. We demonstrate that the substitution of the putative phosphorylation site of caspase-2, Serine-384 to Alanine, blocks caspase-2 processing and decreases its enzymatic activity. Strikingly, in silico analysis using molecular dynamics simulations has shown that Serine-384 is crucially involved in interactions within the caspase-2 active center. It stabilizes Arginine-378, which forms a crucial hydrogen bond with the aspartate residue of a substrate. Hence, Serine-384 is essential for supporting a proper architecture of the active center of caspase-2. Moreover, molecular modeling strongly proved steric inaccessibility of Ser-384 to be phosphorylated. Importantly, a multiple alignment has demonstrated that both Serine-384 and Arg-378 residues are highly conservative across all members of caspase family, which allows us to suggest that this diade is indispensable for caspase processing and activity. Spontaneous mutations in this diade might influence oncosuppressive function of caspases, in particular of caspase-2. Likewise, the mutation of Ser-384 is associated with the development of lung squamous cell carcinoma and adenocarcinoma. Taken together, we have uncovered a central feature of the caspase-2 activation mechanism which is crucial for the regulation of its signaling network.

## Introduction

Apoptosis is an evolutionarily conserved program that regulates the homeostasis and development of multicellular organisms. A number of factors, such as γ-irradiation, DNA damaging agents, hypoxic conditions, deprivation of growth factors or death receptor activation, induce apoptotic cell death^1,2^. Upon DNA damage, cells could undergo apoptosis via the death receptor and/or the intrinsic mitochondrial pathway^3^.

The initiatory and executory roles in apoptosis belong to a family of Cysteine-dependent ASPartate ProteASES (Caspases)^4^. The apoptotic caspases are classified into initiators (caspase-2, −8, −9, and −10) and effectors (caspase-3, −6, and −7), depending on their role in the caspase cascade and their domain structure. Upon cell death signals, the initiator caspases are recruited to high molecular weight platforms and autocatalytically cleaved to form an active zymogen^5^. Once activated, initiator caspases induce effectors to cleave multiple cellular substrates^2,4,6,7^.

Caspase-2 is an evolutionarily conserved member of the family and possesses several unique characteristics, including the presence of a nuclear localization signal, which plays a central role in the triggering of apoptotic pathways after DNA damage in some cellular models. In addition, caspase-2 has been reported to be indispensable for the proper cell proliferation, genomic stability, response to ER and oxidative stress^8^. Moreover, this protein negatively regulates necroptosis and performs a number of non-apoptotic functions^9,10^. Caspase-2, as one of the initiator caspases, has a long prodomain which contains a caspase recruitment domain (CARD). The CARD is followed by two catalytic domains, p19 and p12, which are essential for the formation of caspase-2 active heterotetramer. In response to genotoxic stress, mitotic catastrophe, heat shock, ER stress or bacterial toxins, procaspase-2 has been reported to undergo autocatalytic activation and autoprocessing.

There are many regulatory circuits which tightly control caspase-2 processing and activity. The important and still poorly understood regulatory mechanism of activation of caspases is phosphorylation. The addition and/or removal of the phospho-group induces conformational changes throughout the protein structure or alters the catalytic activity of caspases influencing the cell death program. To date, several phosphorylation sites controlling caspase-2 activity are described^11^.

Upon induction of death receptor-mediated apoptosis, caspase-2 activity is reported to be tightly controlled by the protein kinase CK2, which prevents apoptotic cell death through the phosphorylation of caspase-2 at Ser-157^12^. In metabolic pathways, induction of the pentose phosphate pathway leads to calcium/calmodulin-dependent kinase II (CaMKII) activation and the inhibition of caspase-2 by phosphorylation at Ser-135 (Ser-164 in humans)^13^. During mitosis, the activation of caspase-2 is controlled by another kinase to prevent accidental cell death induction. In particular, caspase-2 is phosphorylated at the conserved position Ser-340 by Cdk1-Cyclin B1 and inhibits enzyme activation^14^. Thus, the phosphorylation/dephosphorylation status of caspase-2 plays a crucial role in the control of cell death or survival programs.

Here, we initially aimed systematically analyze the caspase-2 phosphorylation sites. Using a bioinformatics analysis, we predicted a set of potential phosphorylation sites. Furthermore, using mutagenesis and several biochemical assays, we have shown an importance of Ser-384 for caspase-2 processing and activation. Strikingly, mass-spectrometry analysis revealed that this residue does not undergo phosphorylation. Furthermore, molecular dynamics has demonstrated that Ser-384 plays a crucial role in the stabilization of a key residue of the active center—Arg-378—and is, therefore, essential for substrate binding. However, this amino acid residue locates in a pocket that makes it inaccessible for phosphorylation. Taken together, we found the unique and highly conserved mechanism of caspase activity control and demonstrated its importance for apoptotic cell death.

## Results

### Serine-384 is essential for caspase-2 proteolysis

**Fig. 1:**
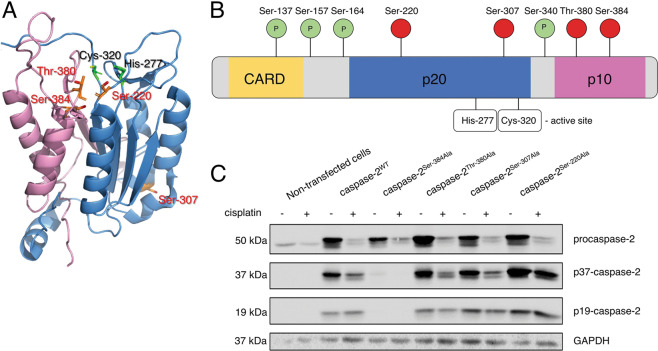
Location of potential phosphorylation sites of caspase-2 in primary and tertiary structure and the role of Ser-384 for caspase-2 processing. **a** The three-dimensional structure of catalytically active fragments of caspase-2 (1pyo.pdb) and potential phosphorylation sites (Ser-307, Ser-220, Thr-380, Ser-384). Predicted phosphorylation sites are highlighted by orange and red, the active center of caspase-2 is shown in green. The large catalytic p19 subunit is shown in blue, and the small catalytic subunit is shown in purple. **b** The reported phosphorylation sites of caspase-2 (green) and potential phosphorylation residues (red) are shown along with two critical caspase-2 active site residues: cysteine (Cys) and histidine (His). **c** The overexpression of caspase-2^WT^ and caspase-2 mutants in HEK293T cells. The processing of caspase-2 in HEK293T cells treated with 70 µM cisplatin for 18 h was analyzed by western blot. The bands corresponding to cleaved fragments of caspase-2 are marked as p37-caspase-2, p19-caspase-2. One representative experiment from three independent experiments is shown.

To gain new insights into the role of caspase-2 phosphorylation in its activation, the systematic prediction of potential phosphorylation sites was carried out. To this point, several bioinformatic algorithms were used. The NetPhos, NetPhorest, ScanSite and PhosphoNet programs identified novel phosphorylation sites by searching common prevalent features of the sequences surrounding the phospho-acceptor residue (Ser/Thr/Tyr) of known phosphoproteins. Based on the highest score results, we selected several residues (Ser-220, Ser-307, Thr-380, Ser-384) for further investigation (Fig. S1). Interestingly, three of them are located in close proximity to the active center of caspase-2 (Fig. 1a, b) and could, therefore, potentially influence caspase-2 activation.

To verify the results of predictions in silico, mutants of caspase-2 (Ser-220Ala, Ser-307Ala, Thr-380Ala, Ser-384Ala) were generated by the substitution of Serine/Threonine residues to Alanine. The caspase-2 mutants and non-mutated caspase-2 (Caspase-2^WT^) were transiently overexpressed in the human embryonic kidney cell line HEK293T. The overexpression of caspases leads to their autocatalytic processing. Accordingly, the overexpression of caspase-2^WT^ as well as of caspase-2^Ser-220Ala^, caspase-2^Ser-307Ala^, and caspase-2^Thr-380Ala^ was sufficient to induce their autocatalytic processing to cleavage products p37 and p19 and generation of caspase-2 catalytic activity (Fig. 1c and S2). To further increase procaspase-2 processing the cells were treated with cisplatin. This led to increased processing of caspase-2^WT^ as well as of caspase-2^Ser-220Ala^, caspase-2^Ser307Ala^, and caspase-2^Thr-380Ala^ and more pronounced decrease of procaspase-2 levels. Importantly, the mutation Ser-384Ala almost completely blocked the processing of caspase-2. In particular, the accumulation of p37 and p19 was strongly decreased, both in the presence and absence of cisplatin treatment (Fig. 1c). Taken together, only mutation of Ser-384 but not Ser-220, Ser-307 or Thr-380 resulted in the inhibition of caspase-2 proteolysis. Therefore, we then aimed to uncover the mechanisms of this phenomenon.

### The Ser-384Ala substitution decreases caspase-2 activity and apoptotic cell death

Next, we studied the effect of the Ser-384Ala substitution of caspase-2 on apoptotic cell death. To this point, we used HEK293T cells and ovarian cancer CAOV-4 cells, in which caspase-2 plays an essential role in DNA damage-induced apoptosis. Indeed, in caspase-2 KO mice ovarian cells are resistant to treatment with DNA damaging agents^15^. Using CAOV-4-shRNA-caspase-2 and CRISPR/Cas9 caspase-2^−/−^ cells (CAOV-4-C2^−/−^) generated by us we demonstrated a significant decrease in caspase-3 processing and the cleavage of PARP in cells with downregulated level of caspase-2 upon cisplatin treatment and apoptosis induction (Fig. S3). Additionally, the cisplatin treatment led to accumulation of p19 fragment and caspase-2 activation in wild type CAOV-4 cells. According to these data, for the next experiments we used this DNA-damaging agent as driver of caspase-2 activation.

**Fig. 2:**
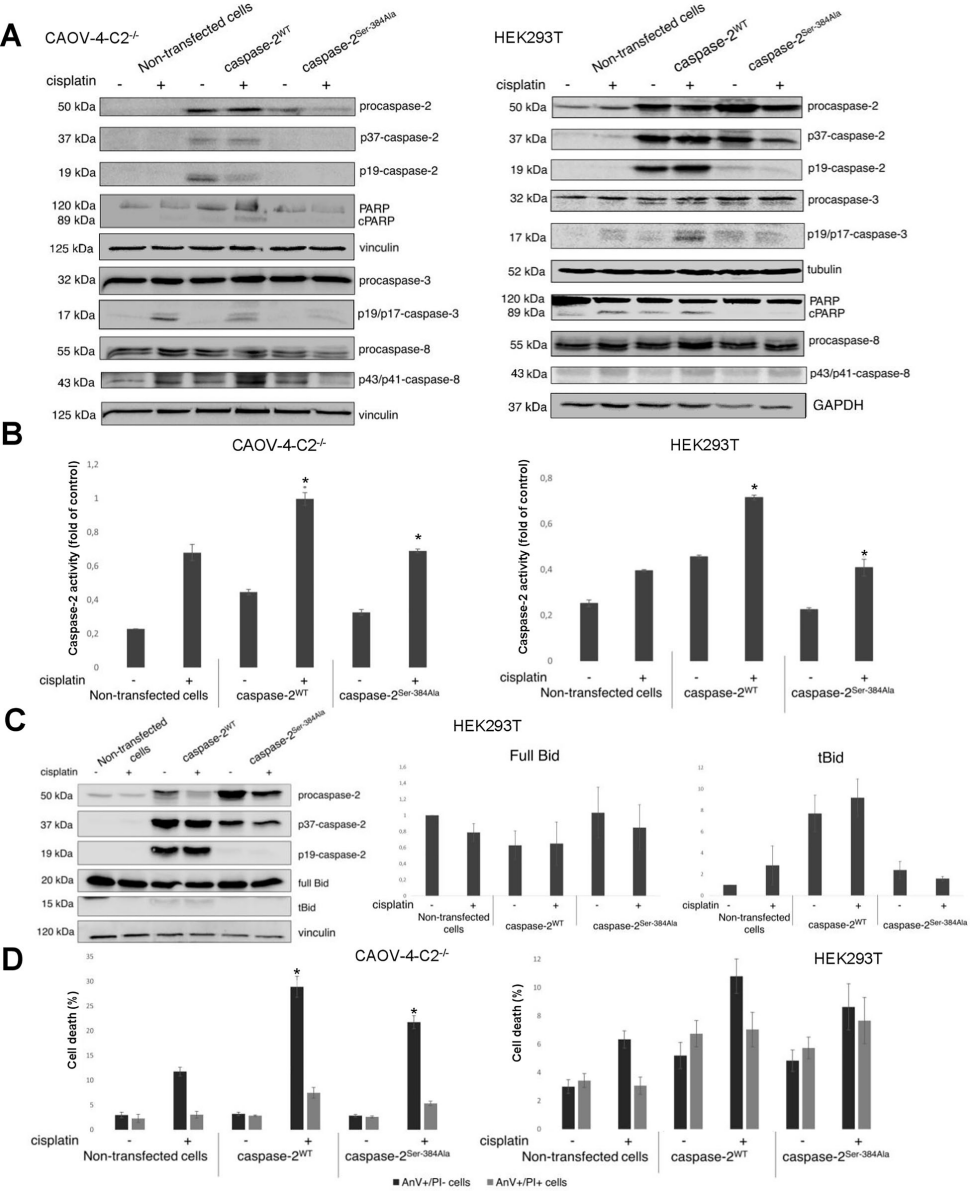
The substitution Ser-384Ala in caspase-2 negatively regulates apoptotic cell death and caspase-2 activity. **a** The processing of caspase-2 and caspase-3 and the appearance of PARP cleavage product p89 (cPARP) in HEK293T and CAOV-4-C2^−/−^ cells that transiently overexpress the caspase-2^WT^ and caspase-2^Ser-384Ala^. Both cell lines were treated with 70 µM cisplatin for 18 h and caspase processing was analyzed by western blot. The bands corresponding to cleaved fragments of caspase-2, caspase-3, caspase-8 and PARP are marked as p37-caspase-2/p19-caspase-2, p19/17-caspase-3, p43/41-caspase-8 and cPARP, correspondingly. **b** The Ser-384Ala substitution led to a reduction of caspase-2 peptidase activity. Caspase-2 activity was measured using a substrate—Ac-VDVAD-AMC in lysates of HEK293T and CAOV-4-C2^−/−^ cells that were non-transfected or transfected with caspase-2^WT^ and caspase-2^Ser-384Ala^. Both cell lines were treated with 70µM cisplatin for 18h and caspase activity was measured. The histograms demonstrate the slope of enzymatic activity curve. **c** The processing of caspase-2 and the cleavage of substrate Bid in HEK293T cells that transiently overexpress the caspase-2^WT^ and caspase-2^Ser-384Ala^. Cells were treated with 70 µM cisplatin for 18 h and caspase processing and Bid cleavage was analyzed by western blot. The bands corresponding to cleaved fragments of caspase-2 are marked as p37-caspase-2/p19-caspase-2, and the truncated form of Bid is marked as tBid. The amount of full-length Bid and tBid forms were quantified by densitometry analysis and normalized on vinculin level. **d** The substitution Ser-384Ala in caspase-2 negatively regulated apoptotic cell death. HEK293T and CAOV-4-C2^−/−^ cells were transfected with caspase-2^WT^ and caspase-2^Ser-384Ala^ and treated for 18h with 70µM cisplatin. Cells were stained with the conjugate of annexin V-FITC (AnV) and propidium iodide (PI) and examined by flow cytometry. The “black” columns demonstrate the AnV+/PI− stained apoptotic population, while the “gray” columns indicate the AnV+/PI+ stained late apoptotic/necrotic cells. Results are presented as the mean of three distinct experiments ± SEM.

For transient overexpression CAOV-4-C2^−/−^ cells were used to minimize the contribution of endogenous caspase-2. The overexpression of caspase-2^WT^ in HEK293T and CAOV-4-C2^−/−^ cells led to its proteolysis in both cell lines upon genotoxic stress, as well as without cisplatin treatment. On the contrary, the overexpression of caspase-2^Ser-384Ala^ demonstrated minor caspase-2 processing in HEK293T cells compared to the overexpression of caspase-2^WT^. Moreover, the complete absence of catalytic fragments p37 and p19 in CAOV-4-C2^−/−^ cells was observed upon caspase-2^Ser-384Ala^ overexpression (Fig. 2a). Apparently, the presence of endogenous caspase-2 in HEK293T might lead to the marginal caspase-2 processing observed in the above-mentioned experiments. Furthermore, caspase-8 and caspase-3 processing with the formation of p19/p17-caspase-3 and the cleavage of PARP were detected upon the overexpression of caspase-2^WT^ in both cell lines (Fig. 2a and S4). In line with the inhibition of caspase-2^Ser-384Ala^ processing, the cleavage of caspase-8, caspase-3 and PARP was largely reduced upon overexpression of caspase-2^Ser-384Ala^. Thus, these data suggested the importance of Ser-384 for caspase-2 activation.

To further investigate the effect of Ser-384 substitution to Ala on caspase-2 activation, we measured the catalytic activity of caspase-2 using fluorescent substrate VDVAD-MCA in HEK293T and CAOV-4-C2^−/−^ cells that transiently overexpressed the caspase-2^WT^ and caspase-2^Ser-384Ala^. The overexpression of the caspase-2^WT^ led to increased caspase-2 activity in both cell lines accompanied by a strong enhancement upon cisplatin treatment (Fig. 2b). Importantly, the overexpression of caspase-2^Ser-384Ala^ did not increase caspase-2 activity, further supporting the inhibitory effects of Ser-384 substitution to Ala. Importantly, the level of enzyme activity was equal to the background level in non-transfected samples. It should be added that the appearance of caspase-2-like activity in non-transfected CAOV-4 KO-caspase-2 cells could be explained by the presence of other proteases with VDVAD-like cleaving enzyme activity^16^.

Moreover, we analyzed the cleavage of caspase-2 intracellular substrate Bid in HEK293T cells that transiently overexpressed the caspase-2^WT^ and caspase-2^Ser-384Ala^. The western blot results clearly demonstrated the absence of p19-fragment of caspase-2 in samples with the mutant form of caspase-2 and the level of full-length Bid comparable to the untransfected cells (Fig. 2с). The overexpression of the caspase-2^WT^ led to increased caspase-2 processing and the appearance of truncated form of Bid and was more pronounced upon cisplatin treatment. Thus, the Ser-384 indeed is essential for caspase-2 processing and enzymatic activity.

Furthermore, we studied the effect of Ser-384Ala substitution on apoptotic cell death by Annexin V/Propidium iodide staining (AnV/PI) in combination with flow cytometry. The flow cytometry analysis elucidated that cisplatin treatment led to increased apoptosis induction in both cell lines (Fig. 2d). The overexpression of caspase-2^WT^ enhanced apoptotic cell death upon genotoxic stress in these cell lines. Interestingly, the overexpression of caspase-2^Ser-384Ala^ inhibited cisplatin-induced apoptosis compared to the overexpression of caspase-2^WT^. These results further confirmed the importance of Ser-384 for caspase-2 activation and cell death. Taken together, the caspase-2^Ser-384Ala^ did not possess the ability to undergo processing and activation. Moreover, the overexpression of this mutant inhibited cisplatin-induced cell death in comparison to caspase-2^WT^.

### The Ser-384 of caspase-2 was not found to be phosphorylated

**Fig. 3:**
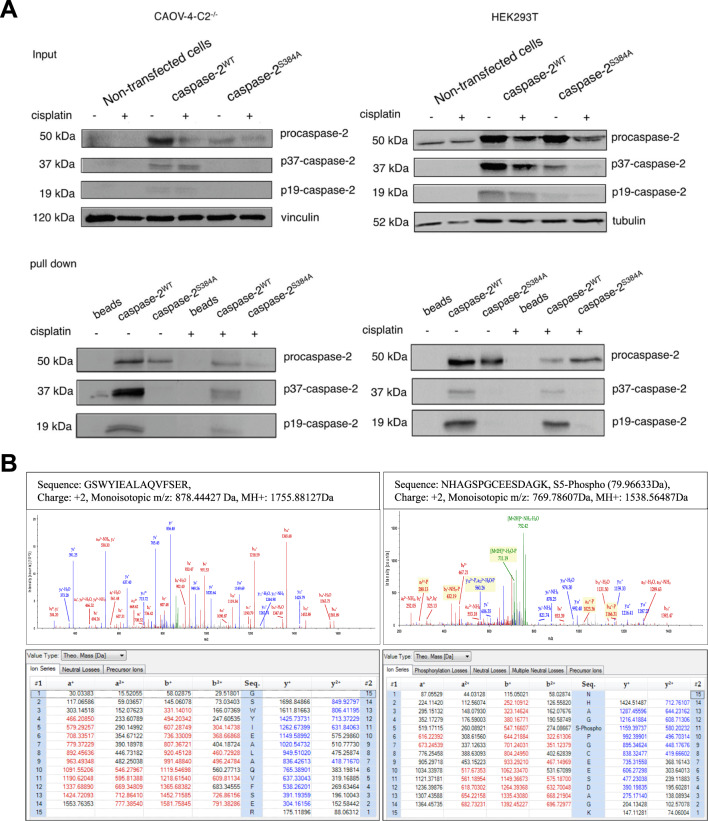
The pull-down assay of caspase-2WT and mutant form (Ser-384Ala) transiently overexpressed in HEK293T and CAOV-4-C2-/- cells and LC-MSMS analysis. **a** The processing of caspase-2 was analyzed by western blot. The bands corresponding to cleaved fragments of caspase-2 are marked as p37-caspase-2/p19-caspase-2. **b** Annotated spectra are shown with all of the fragments that matched an expected fragment from in silico digestion (in color). Below each spectrum is a table with calculated ion masses for each peptide; colored values match corresponding peaks in the spectrum.

Next, we investigated whether caspase-2 is phosphorylated at Ser-384 in the presence or absence of cisplatin treatment. We decided to use this compound since as mentioned above cisplatin triggers caspase-2 activation in CAOV-4 cells (Fig. S3) and does not do that in ovarian cells isolated from caspase-2-null mice^15^. To address this point, we overexpressed the Strep-tagged-caspase-2^WT^ and the Strep-tagged-caspase-2^Ser-384Ala^ in HEK293T and CAOV-4-C2^−/−^ cells. The pull-down experiments clearly demonstrated the absence of any cleavage fragments of caspase-2 in samples with the mutant form of caspase-2 in both cell lines that confirmed our previous results (Fig. 3a). Then, we performed mass-spectrometry (MS) analysis of the obtained samples to evaluate the potential phosphorylation sites of caspase-2. Interestingly, the MS/MS analysis detected a phosphopeptide, NHAGsPGCEESDAGK, with phosphorylation on the first Serine corresponding to Ser-340 in caspase-2. This phosphopeptide was reproducibly detected in both untreated and cisplatin-treated samples (Fig. 3b). Notably, Ser-340 phosphorylation has been shown previously, which confirmed the reliability of the approach used^14^. However, the phosphorylation at Ser**-**384 was not detected (peptide GSWYIEALAQVFSER (Fig. 3b)). Further, neither the phosphorylation of Ser-157 (peptide LSTDTVEHSLDNK (Fig. S5)), nor phosphorylation at Ser-220 (SGGDVDHSTLVTLFK (Fig. S5)) were detected with LC-MSMS analysis. Thus, using mass-spectrometry approaches we could not identify the phosphorylation of Ser-384.

**Fig. 4:**
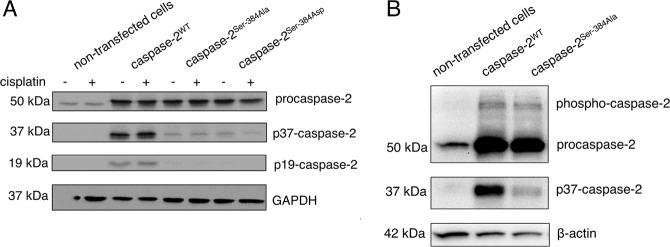
Phospho-mimetic mutation and Phos-tag gel electrophoresis cannot reveal Ser-384 phosphorylation. **a** The processing of caspase-2 in HEK293T cells, which transiently overexpressed caspase-2^WT^, caspase-2^Ser-384Asp^, and caspase-2^Ser-384Ala^ (Ser-384Ala, Ser-384Asp), and were treated with 70 µM cisplatin for 18 h, was analyzed by western blot. The bands corresponding to cleaved fragments of caspase-2 are marked as p37-caspase-2/p19-caspase-2. **b** The detection of phosphorylated caspase-2 by Phos-tag SDS-PAGE in combination with western blot. The lysates from HEK293T cells with transiently overexpressed caspase-2^WT^ and caspase-2^Ser-384Ala^ were subjected to the analysis. The bands corresponding to caspase-2 and its predicted phosphoforms are shown.

To validate the mass-spectrometry results, a phospho-mimetic mutant was constructed by the substitution of Ser-384 with Aspartate (Asp). As shown in Fig. 4a, the overexpression of both caspase-2^Ser-384Asp^ and caspase-2^Ser-384Ala^ in HEK293T cells demonstrated a decrease in the processing of caspase-2 in comparison to caspase-2^WT^ (Fig. 4a). Importantly, regardless of genotoxic stress, the substitution at the Ser-384 site with a negatively charged amino acid did not restore caspase-2 activation, similar to that seen for the Ser-384Ala substitution. To further validate these results, the phosphate-affinity polyacrylamide gel electrophoresis (Phos-tag gel electrophoresis) in order to detect mobility shifts corresponding to phosphorylated caspase-2 forms was performed. Interestingly, the overexpression of caspase-2^WT^ and caspase-2^Ser-384Ala^ induces the appearance of several bands on western blot that correspond to non-phosphorylated and phosphorylated forms of caspase-2. However, no difference in the phosphorylation pattern of caspase-2^WT^ versus caspase-2^Ser-384Ala^ was observed (Fig. 4b). Thus, we can conclude that caspase-2 could be phosphorylated at Ser-340, as shown by mass-spectrometry analysis, but was not phosphorylated at Ser-384.

### The hydroxyl group of Ser-384 is crucial for substrate binding to Arg-378 in the active center of the caspase-2 enzyme

**Fig. 5:**
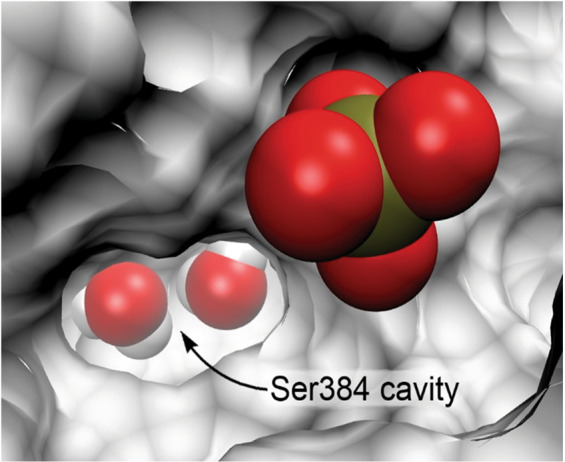
The Ser-384 cavity in the active site of caspase-2 (apo-form). Water molecules are shown inside the cavity, and a model phosphate group is shown at the entrance to the cavity. The narrow entrance does not allow the bulky phosphate group to enter the cavity and get closer to the Ser-384 side chain.

To study the mechanism of the negative effect of Ser-384Ala substitution on processing and enzymatic activity of caspase-2, we analyzed the spatial location of the Ser-384 residue in the active center using the available 3D-structure of caspase-2 (1pyo.pdb)^17^. Ser-384 is located at the bottom of a small cavity in the active site, known as the S_1_ specificity pocket, and its side chain is involved in the formation of the cavity’s surface together with the Arg-219 and Arg-378 side chains. The carboxyl group of the substrate’s aspartate residue (D1) binds in the S_1_ pocket, which is critical for substrate recognition^17,18^. First, we focused on modeling the putative phosphorylation of Ser-384 in silico. Strikingly, it was revealed that the phosphate group is too bulky to enter the Ser-384 cavity and covalently bind to the Ser-384 side chain. This is shown in Fig. 5, using a solid protein surface representation and a model of phosphate ion. The cavity entrance could be described as an ellipse with axes of ≈6.2 and 3.5 Å, which fits very well the planar structure of the substrate’s carboxyl group but is extremely narrow for accommodating the tetrahedral phosphate group (geometric characteristics of the carboxylate and phosphate groups are given in Fig. S6).

**Fig. 6:**
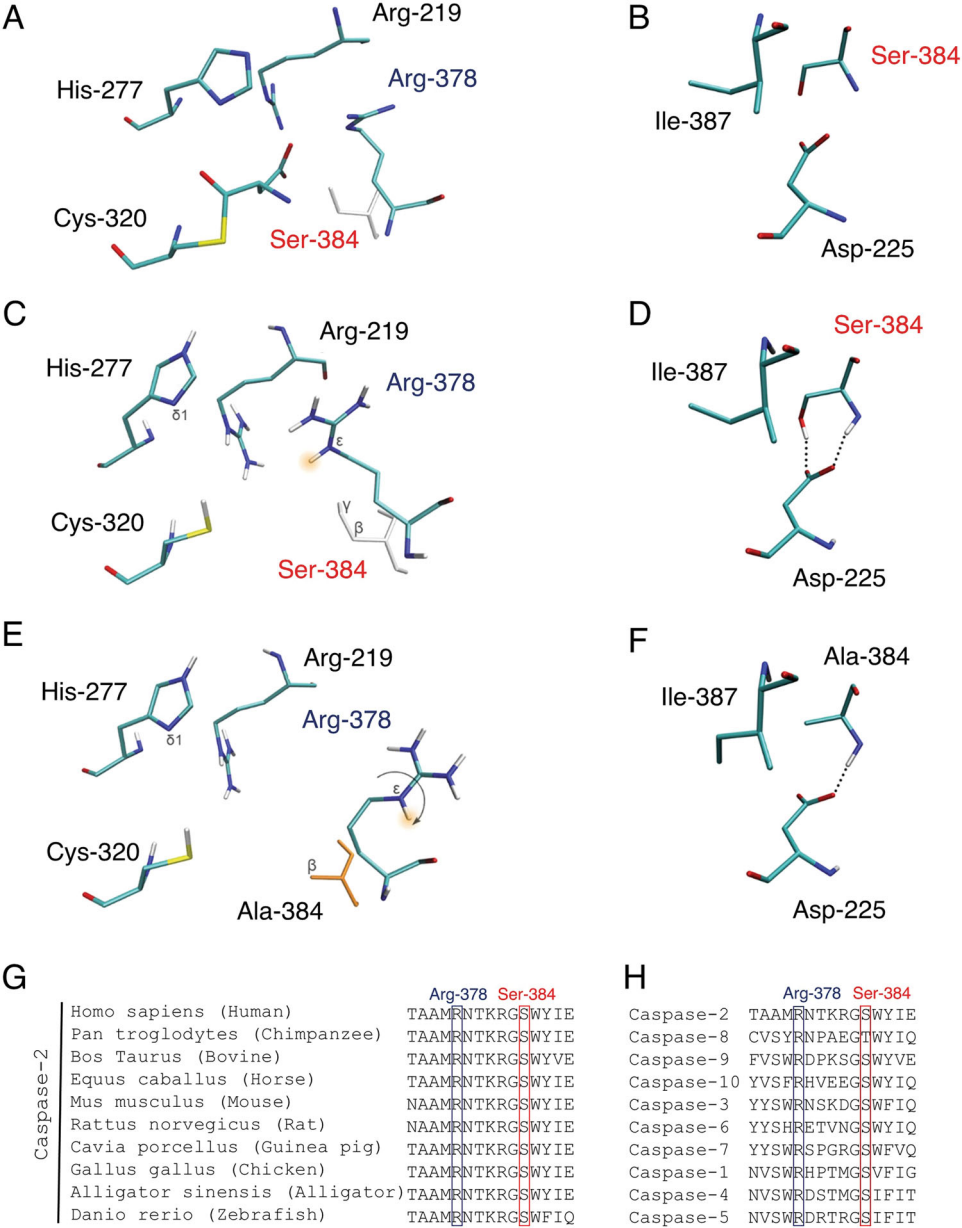
The conservative role of Ser-384 residue in the stabilization of Arg-378 and substrate recognition of caspase-2. **a**, **b** The crystal structure of a complex with substrate analogue (Cys-320 is covalently bound to the aspartate residue; PDB ID 1pyo). **c**, **d** The MD model of the native apo form. **e**, **f** The MD model of the Ser-384Ala apo-form. **a**, **c**, **e** Give an overall view of the active site, while **b**, **d**, **f** show the local environment of the Ser-384/Ala-384 residue. The arrow shows the conformational change induced by the Ser-384Ala mutation. **g**, **h** Multiple alignment (ClustalO) demonstrating the conserved Ser-384 and Arg-378 involved in the substrate binding in the caspase family and across different species. The blue and red frames show the highly conserved residues Arg-378 and Ser-384, respectively.

Next, we hypothesized that one of the most likely reason leading to the disturbance of caspase-2 activity could be the loss of the ability to bind the substrate molecule. The S_1_-pocket residues Arg-219 and Arg-378 form hydrogen bonds with the aspartate D1 while Ser-384 does not mediate direct interactions with the substrate (Fig. 6a). Instead, Ser-384 forms hydrogen bonds with the Asp-225 side chain (Fig. 6b). Thus, the Ser-384Ala substitution does not seem to produce new direct interactions with the substrate but could influence the structure of the caspase-2 active site.

To confirm this hypothesis, we applied Molecular Dynamics (MD) modeling approaches. MD modeling allowed us to compare and analyze the structures of the active center of caspase-2^WT^ and caspase-2^Ser-384Ala^. Upon mutation of Ser-384 to Ala, this amino acid loses one of the hydrogen bonds with Asp-225 and forms a hydrophobic contact with the Ile-387 side chain (Fig. 6d, f). However, the conformation of the Asp-225 and Ile-387 residues does not change significantly. Among the key residues involved in substrate binding, Arg-378 undergoes a major conformational change: the guanidinium group turns so that the N^ε^ atom loses the ability to form an important hydrogen bond with the substrate’s aspartate residue (Fig. 6c, e). Part of the MD trajectory, where the transition of Arg-378 to the non-productive conformation occurs, is shown in the Supplementary Movie. In both the native and mutant forms of caspase-2, the SH-group of catalytic Cys-320 is oriented directly towards the N^δ1^ atom of His-227 (Fig. 6c, e), which is consistent with the mechanism of caspases action where the cysteine acts as the nucleophile and histidine as the general base^19^. The Ser-384Ala substitution in caspase-2 does not affect the catalytic dyad His-227 to Cys-320, but apparently disrupts the binding of the substrate to Arg-378. The mutation prevents the electrostatic interaction between the O^γ^ atom of Ser-384 (partial negative charge) and Arg-378 guanidinium group (positive charge) and induces the conformational change of Arg-378 with the formation of compensatory hydrogen bonds between the guanidinium group and the bulk solvent. Additionally, a multiple alignment of the primary structure of initiator, executor and inflammatory caspases and comparison of their tertiary structure demonstrated a high conservation of both Ser-384 and Arg-378 residues across the caspase family (Fig. 6h) and across different species (Fig. 6g). This analysis supports an important role of the Ser-384/Arg-378 interaction for the proper molecular architecture of the active center of caspase-2. Taken together, these results clearly showed that the Ser-384 residue plays a crucial role in substrate binding and is necessary for caspase-2 processing and enzymatic activity to induce apoptotic cell death.

## Discussion

In this study, we have revealed a conservative and important role of the Ser-384 residue in the formation of the cavity’s surface at the caspase-2 active center. These findings highlight its role in substrate recognition and enzyme activity. Moreover, this analysis allowed us to gain new insights into the molecular architecture of the active center of caspase-2 and uncover a new set of regulatory interactions that might play an essential role in other caspases.

Strikingly, at first, we presumed that Ser-384 and other predicted residues could be phosphorylated and take part in caspase-2 activation control. Notably, the ScanSite and NetPhos programs that were used for the prediction of modification sites have already been successfully applied for the prediction of caspase phosphorylation^20–23^. Using site-directed mutagenesis (Ser/Thr to Ala) of the selected phosphorylation sites and biochemical techniques, we demonstrated that only the mutation of Ser-384Ala prevented caspase-2 processing and blocked enzymatic activity. Moreover, we found that the mutation of Ser-384 independent of the endogenous levels of caspase-2 impaired caspase-2 activation and the induction of apoptotic cell death. Notably, caspase-2^Ser-384Ala^ might potentially form a dimer with caspase-2^WT^, but this dimerization does not seem to allow the formation of active enzymes because the overexpression of caspase-2^Ser-384Ala^ fully prevented an increase in fluorogenic substrate cleavage. Our observations were confirmed by the fact that the cleavage of Bid, one of the important caspase-2 substrates, was significantly decreased in the cells overexpressed caspase-2 Ser-384Ala mutant.

As was mentioned above, the Ser-384 residue is located in close proximity to the active center of caspase-2 at the same spatial region of the alpha-helix in the large catalytic subunit as Thr-263 of caspase-8 and Ser-183 of caspase-9. The phosphorylation of the latter residues has already been shown^24,25^. To confirm our hypothesis regarding Ser-384 phosphorylation, we performed mass-spectrometry analysis of caspase-2 and generated a phospho-mimetic mutant to imitate caspase-2 phosphorylation. Strikingly, we could not identify significant changes in caspase-2 processing in phospho-mimetic form or find any phosphopeptide of Ser-384 by MS/MS analysis. However, the mass-spectrometry analysis demonstrated phosphorylation of the Ser-340 residue in accordance with the previous publications. In previous studies, it was shown that caspase-2 is phosphorylated at Ser-340 by Cdk1-Cyclin B1 during mitosis to prevent apoptotic cell death that is reversed by the action of PP1 during interphase^14^.

To elucidate the molecular mechanism by which Ser-384 supports caspase-2 processing and activity, we performed MD of the three-dimensional structure of the apo-form of caspase-2. Using this approach, we demonstrated that the disturbance of caspase-2 activity and processing caused by Ser-384 substitution might result from the loss of the ability to bind the substrate molecule. Interestingly, Ser-384 does not affect the catalytic dyad His-227/Cys-320 or directly interact with the substrate but is involved in electrostatic interactions with Arg-378, which in turn forms a hydrogen bond with the substrate aspartate residue. Moreover, Tang et al. clearly demonstrated the importance of the Arg-378 residue location and its role in caspase-2 activation by crystallization methods^18^. As described above, the discovered mechanism of substrate molecular recognition and stabilization by Arg-378 and Ser-384 is strongly conservative across all members of the caspase family and seemingly plays a crucial role in caspase activity

Recently, the data describing the role of Ser-384 in caspase-2 activation were reported^26^. The eleven putative sites of caspase-2 phosphorylation were detected and analyzed in U2OS cells applying MS approach. Using phospho-mimetic substitutions, it was demonstrated that only Ser-384Glu substitution blocks caspase-2 processing and suppresses substrate cleavage. According to our results, this mutant also leads to the abrogation of the architecture of the caspase-2 active center via perturbing the interactions of Ser-Arg diade. In addition, the authors have suggested that Ser-384Glu mimics caspase-2 phosphorylation that negatively regulates activation of the protein^26^. In contrast to these data, we demonstrated that Ser-384Ala substitution has the same effect and blocks caspase-2 activity. In case of phosphorylation, the mutation Ser-384Ala should withdraw the negative regulation of this modification and promote caspase-2 processing and activation. According to MD of the three-dimensional structure of caspase-2 the loss of O^γ^ atom of Ser-384 induces the conformational change of Arg-378 and disturbs the proper molecular architecture of the active center of caspase-2. Consequently, the substitution of Ser-384 with Ala has the same negative effect on caspase-2 activation as the replacement of phosphomimetic amino acid residue (Glu or Asp) because any substations disrupt the interaction between Ser-384 and Arg-378. Moreover, the modeling of the putative phosphorylation of Ser-384 in silico revealed that the phosphate group is too bulky to enter the Ser-384 cavity and covalently bind to the Ser-384 side chain. Hence, in fact the data from Lim et al. tailored (or corroborate, or agreed) our results although we completely exclude the possibility of Ser-384 phosphorylation^26^.

Another fascinating feature that we derived from the TCGA database analysis is the presence of missense and stop-gain mutations in caspase-2/−3/−6 at a conserved arginine position, which corresponds to the Arg-378 residue in caspase-2 (Data generated by the TCGA Research Network: https://www.cancer.gov/tcga). The Arg-378Trp mutation in caspase-2, or Arg-207Stop in caspase-3, or Arg-220Trp in caspase-6 were found in uterine corpus endometrial carcinoma and is probably involved in apoptotic cell death disturbances and tumorogenesis. Interestingly, the Serine conserved position that corresponds to the Ser-384 residue in caspase-2 is also subjected to missense mutagenesis in caspase-5 and -6 in lung squamous cell carcinoma and adenocarcinoma.

Thus, the obtained results demonstrate the crucial role of the Ser-384 and Arg-378 residues in substrate binding, caspase-2 processing, and enzymatic activity. Moreover, the described mechanism is conserved and probably common for other members of the caspase family. The mutations of these residues were found in endometrial carcinoma and lung cancer. In this regard, our findings are crucial for obtaining new insights into regulatory mechanisms of apoptotic cell death and associated disorders as well as for the development of new effective anticancer therapies and diagnostic tests.

## Materials and methods

### Cell culture and treatment

Parental CAOV-4 and HEK293T cells were obtained from the ATCC and a gift from the Division of Toxicology, Karolinska Institute (Stockholm, Sweden). CRISPR/Cas9 KO caspase-2 CAOV4 cells were generated as described below. CAOV-4 cells were transfected with control CRISPR/Cas9 (sc-418922) or caspase-2 CRISPR/Cas9 KO (sc-401079, both from Santa Cruz Biotechnology, USA) plasmids. All cell cultures were maintained in a CO_2_ incubator (5% CO_2_) in DMEM medium, containing 10% fetal bovine serum at 37 °C, in the presence of 1% penicillin/streptomycin (Merck Millipore, USA). Cells in the logarithmic growth phase were used for experiments. To trigger apoptosis, cells were stimulated with 70 μM cisplatin (Teva, Israel) for 18 h.

### CRISPR/Cas9 transfection

CAOV-4 cells were transfected with Caspase-2 CRISPR/Cas9 KO plasmids using Lipofectamine™ LTX Reagent with PLUS™ transfection reagent (#15338100, ThermoFisher, USA) according to manufacturer’s protocol. The knockout plasmids sc-401079 (Santa Cruz, USA) are a mixture of three plasmids, each carrying a different guide RNA specific for the target gene, as well as the Cas- and GFP-coding regions. GFP-positive cells were selected by cell sorting on a FACSAria III Cell Sorter (BD Bioscienecs, USA) after 24 h of transfection. The depletion of the target protein was verified by western blot.

### Flow Cytometry analysis

Cells were incubated in Petri dishes as indicated with or without cisplatin, then collected by 0.05% trypsin-EDTA (Life Technologies, Germany) and transferred to a conditioned medium. The cells were centrifuged (2000 rpm, 5 min, 4 °C) and washed twice with cold PBS (Life Technologies, Germany). Then, cells (0.2×10^6^) were resuspended in 200 µl Annexin-binding buffer (#556454, BD Biosciences, USA) and 2 µl of Annexin-V-FITC (A13199, Invitrogen, USA) were added to the sample and incubated in the dark at 4 °C for 15 min. Next, 5 µl propidium iodide (50 µg/ml) (#556463, BD Biosciences, USA) was added to each sample. After incubation for five minutes in the dark at room temperature, the cells were analyzed by flow cytometry BD FACSCANTO II (BD Biosciences, USA).

### Cloning and mutagenesis

#### Cloning experiments

pESG-IBA103 plasmid (IBA-lifesciences, Germany) is selected for highly productive gene expression in mammalian cells and contains Strep-tag for efficient purification through affinity chromatography. Based on the pcDNA3-caspase-2 plasmid, a PCR fragment encoding a full-length caspase-2 sequence was obtained (Forward: AGCGCGTCTCCAATGATGGCGGCGCCGAGCGC, Reverse: AGCGCGTCTCCTCCCTGTGGGAGAGGGTGTCCTGG). A PCR fragment was isolated by Gene JET Gel Extraction kit (Fermentas, USA) and cloned according to the StarGate Direct Transfer Cloning kit (IBA-lifesciences, Germany) into pESG-IBA103 plasmid. *E. coli* XL-1 Blue cells were transformed using a ligase mixture according to a standard technique. The correct sequence of the obtained plasmid DNA was confirmed by sequencing.

#### Mutagenesis

The pESG-IBA103 plasmid containing the *caspase-2* gene that was described above was used for mutagenesis. Forward primers and their reverse complement were designed to replace selected residues in the *caspase-2* gene:

Ser-220Ala (Forward: GAACTGGAATTTCGCGCTGGAGGGGATGTGG, Reverse: CCACATCCCCTCCAGCGCGAAATTCCAGTTC),

Ser-307Ala (Forward: GGTTTGTTCTGTAGGGCTGGGCAGTTGGCGTTGTC, Reverse: GACAACGCCAACTGCCCAGCCCTACAGAACAAACC),

Thr-380Ala (Forward: GGAACCTCGTTTGGCGTTCCGCATGGCGG, Reverse: CCGCCATGCGGAACGCCAAACGAGGTTCC),

Ser-384Ala (Forward: CCTCGATGTACCAGGCACCTCGTTTGGTGTTCC, Reverse: GGAACACCAAACGAGGTGCCTGGTACATCGAGG),

Ser-384Asp (Forward: GAGCCTCGATGTACCAGTCACCTCGTTTGGTGTTCC, Reverse: GGAACACCAAACGAGGTGACTGGTACATCGAGGCTC).

Site-directed mutagenesis was carried out using the Quickchange Site-Directed Mutagenesis kit (Agilent Technologies, USA) according to the manufacturer’s instructions. The correct sequence of the mutants was confirmed by sequencing.

### Transfection

For transfection, pESG-IBA103 caspase-2 wild type/Ser-220Ala/Ser-307Ala/Thr-380Ala/Ser-384Ala/Ser-384Asp plasmids were used. Cells were transfected with the designated plasmids, using Lipofectamine LTX/Plus (Life Technologies, Germany) according to the manufacturer’s instructions. Six hours after transfection, the medium was changed and drugs were administered. Cells were assayed 18 h after drug treatment.

### Measurement of caspase-2 activity

Caspase-2 activity was assessed by detecting the cleavage of fluorogenic peptide substrates VDVAD-AMC (PeptaNova, Germany). Harvested cells were re-suspended with PBS (100 μl PBS per 1 × 10^6^ cells). Then, 25 μl of the suspension was placed into a 96-well plate and mixed with the appropriate peptide substrate (100 μM) dissolved in 50 μl of caspase-2 reaction buffer (100 mM MES, pH 6.5, 10% polyethylene glycol, 5 mM DTT, 0.001% NP-40, 0.1% CHAPS). Cleavage of the fluorogenic peptides was monitored at 37 °C using a VarioScan Flash multimode detector (Thermo Scientific, USA) by AMC liberation at wavelengths of 380 nm for excitation and 460 nm for emission. The fluorescence values were normalized to protein concentrations measured using the Pierce BCA Protein Assay Kit (Thermo Scientific, USA).

### Pull down assay

Total cell lysates were used for the pull-down assay. Here, 50 μl of Strep-Tactin®XT Superflow (IBA Lifesciences, Germany) was added to the 1 ml lysates with a total protein concentration of 3–5 mg/ml and incubated with gentle rotation for 3 h at 4 °C. Then, samples were subjected to centrifugation (3000 rpm, 3 min, 4 °C). The Strep-Tactin®XT beads were washed three times with 1 ml of 10 mM Tris-HCl-buffer, pH 7.4, containing 100 mM NaCl, 1 mM EDTA, 1 mM EGTA, 1% Triton X-100, 1% PMSF, protease inhibitor cocktail (Roche, Switzerland), phosphatase inhibitor cocktail (Merck, USA) and one time with PBS. Then, the beads were dried with a syringe; one part (20%) was analyzed by western blot and the remainder (80%) was subjected to mass-spectrometry.

### Mass-spectrometry analysis

#### Sample-preparation

After affinity chromatography, beads with bound proteins were washed three times with 50 mM ammonium bicarbonate and digested with 0.4 µg trypsin in 100 µL overnight at 37 °C. The supernatant was removed from the beads, and beads were washed with fresh ammonium bicarbonate and later pooled. Samples were desalted on 18C pipette tips (Thermo Scientific, USA) according to the manufacturer’s instructions; the obtained peptides were dried and then dissolved in 0.1% (v/v) formic acid in water prior to LC-MS/MS analyses.

#### LC-MS/MS analysis

Automated online analyses were performed with a LTQ Orbitrap Velos Pro hybrid mass spectrometer (Thermo Scientific, USA) equipped with a nano-electrospray source. On-line reversed-phase liquid chromatography to separate peptides was performed using an EASY-nLC 1200 system (Thermo Scientific, USA). All samples were analyzed on a 20 mm × 100 μm C18 pre column followed by a 100 mm × 75 μm C18 column with a particle size of 5 μm (NanoSeparatoons, Nieuwkoop, Netherlands) at a flow rate of 300 nL/min with EASY-nLC II (Thermo Scientific, USA) using a gradient of Buffer A (0.1% formic acid) and Buffer B (0.1% formic acid in acetonitrile) at a flow rate of 300 nL/min as follows: from 2% B to 40% B in 90 min; from 40% B to 90% B in 20 min, and maintained at 90% B for 10 min. Full scans were performed at a resolution of 60,000; the top 20 most intense multiply charged ions were selected with an isolation window of 2.0 and fragmented in the linear ion trap by collision-induced dissociation with a normalized collision energy of 30%. Dynamic exclusion of sequenced peptides for 60 s and charge state-filtering disqualifying singly charged peptides was activated and predictive automatic gain control (AGC) was enabled.

#### Database search

Raw files were searched using the Sequest HT search algorithm in Proteome Discoverer (Thermo Fisher Scientific, San Jose, CS, USA; version 1.4.0.288) against a Uniprot Human 9606 database (March 2018) with the following parameters: digestion enzyme trypsin; maximum number of missed cleavages 2; fragment ion mass tolerance 0.60 Da; parent ion mass tolerance 10.0 ppm and variable modifications were the oxidation of methionine, phosphorylation of serine, threonine and tyrosine. The fixed value PSM validator search node was used; the results were filtered with a “high” peptide confidence value and a target false discovery rate of 0.05. Manual spectra inspection was conducted on all potential phosphopeptides to eliminate false positives. Each final data set was the result of the three biological replicates

### SDS-PAGE, Phos-tag SDS-PAGE, and western blot

Laemmli’s loading buffer was added to 40 μg of proteins of cell lysates; after boiling for 5 min at 95 °C, samples were subjected to SDS-PAGE or Phos-tag SDS-PAGE. The SDS-polyacrylamide gels consisted of 12% acrylamide separation gel and a 4% stacking gel. The Phos-tag gels consisted of a separating gel copolymerized with Phos-tag (8% acrylamide, 20 μM acrylamide-pendant Phos-tag, 40 μM MnCl_2_). After electrophoresis, Phos-tag gels were soaked in a transfer buffer (25 mM Tris, 192 mM glycine, and 20% ethanol) containing 1 mM EDTA for 20 min with gentle agitation for elimination of the manganese ions from the gel. Next, the gels were soaked in a transfer buffer without EDTA for 10 min with gentle shaking and were blotted onto nitrocellulose membranes using Bio-Rad TransBlot Turbo (Bio-Rad, USA) machine. Then, the membranes were transferred to a solution of 5% non-fatty milk in PBST (PBS + 0.05% Tween-20) and washed in PBST three times for 10 min. The membranes were incubated with primary antibodies against caspase-2 (#611022) and PARP (#611038, both from BD Transduction Lab, USA), caspase-8 (#9746), caspase-3 (#9662, all from Cell Signaling, USA), actin (A2066, Sigma-Aldrich, USA), tubulin (ab7291, Abcam), and GAPDH (#2118, Cell Signaling, USA) overnight at 4 °C. After three washes in PBST, the membranes were incubated with goat anti-rabbit IgG-HRP antibody (ab97200) or with goat anti-mouse IgG-HRP antibody (ab97265, both from Abcam, USA) for one hour. Then, the membranes were washed four times in PBST and developed with ECL (Promega, USA) or SuperSignal West Dura Extended Duration Substrate (Thermo Scientific), and images were acquired with the Imager ChemiDoc™ (Bio-Rad, USA).

### Molecular modeling

The molecular models of native and mutant forms of human caspase-2 were built on the basis of the 1pyo crystal structure^17^. The Ser-384Ala mutant was generated by removing the O^γ^ atom in Ser-384. Hydrogen atoms were added to the protein structure considering the ionization of amino acid residues, and then it was solvated by a 12-Å thick layer of TIP3P water. Sodium ions were added to neutralize the system. For the starting models of caspase-2, an equilibration and subsequent 10 ns MD simulation were carried out according to the following protocol. Initially, the two-stage energy minimization was performed to relax the solvated system. The first stage included 2500 steepest descent steps followed by 2500 conjugate gradient steps with positional restraints on protein atoms. The second stage included 5000 steepest descent steps and 5000 conjugate gradient steps without any restraints. The energy-minimized system was heated up from 0 to 300 K over 50 ps with positional restraints on the protein, and then equilibrated over 500 ps at 300 K. Finally, a 10 ns MD trajectory was simulated (300 K, constant pressure) for subsequent analysis of the protein structure.

The preparation of starting systems and the trajectory analysis were performed using AmberTools 15 (http://ambermd.org); the energy minimization and MD simulations were performed using Amber 14^27^ with the ff14SB force field^28^. VMD 1.9.2^29^ and VideoMach 5.15.1 (http://gromada.com/videomach) were used for the visualization of structures and trajectories.

### Statistical analysis

Data are presented as means ± sem. of at least 3 independent experiments. Statistical analysis was performed using Student’s *t*-tests at a significance level of *p < 0.005.

## Supplementary information

Movie

Supplementary Figure Legends

Supplementary Figure 1

Supplementary Figure 2

Supplementary Figure 3

Supplementary Figure 4

Supplementary Figure 5

Supplementary Figure 6
