# Correction: Requirement for Serine-384 in Caspase-2 processing and activity

**DOI:** 10.1038/s41419-025-08271-y

**Published:** 2025-12-23

**Authors:** Alexey V. Zamaraev, Pavel I. Volik, Dmitry K. Nilov, Maria V. Turkina, Aleksandra Yu. Egorshina, Anna S. Gorbunova, Svetlana Iu. Iarovenko, Boris Zhivotovsky, Gelina S. Kopeina

**Affiliations:** 1 Faculty of Medicine, MV Lomonosov Moscow State University, Moscow, 119991 Russia; 2 Belozersky Institute of Physicochemical Biology, MV Lomonosov Moscow State University, Moscow, 119991 Russia; 3 Faculty of Medicine and Heath Sciences, Department of Clinical and Experimental Medicine, Linköping University, 58185 Linköping, Sweden; 4 Division of Toxicology, Institute of Environmental Medicine, Karolinska Institute, Box 21017177 Stockholm, Sweden

Correction to: *Cell Death & Disease* 10.1038/s41419-020-03023-6, published online 03 October 2020

Figure 2A contains technical errors. In the right panel (HEK293T), the loading control (vinculin) for PARP, p43/p41 caspase-8, and procaspase-8 was used mistakenly. In the new version GAPDH is correctly used as confirmed by shape and form of bands. The molecular weight marker for the p43/p41 caspase-8 bands was incorrectly designated as 18 kDa, although the correct size is 43 kDa. The loading control (vinculin) for caspase-2 and PARP in the left panel, CAOV-4-C2-/-, was previously omitted, which should also be corrected.

We have prepared the corrected version of Figure 2, that addresses these points.

**Figure 2A Original** 
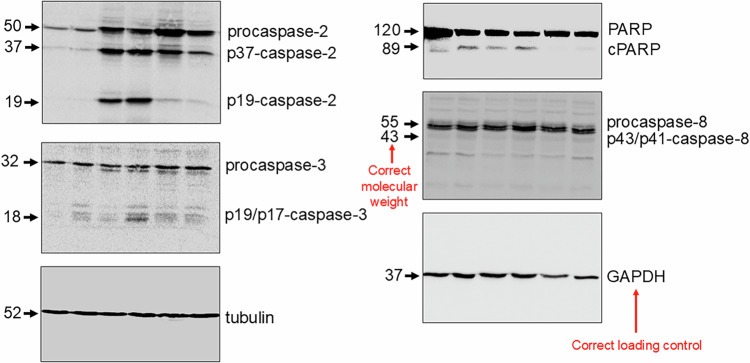


**Figure 2A Corrected** 
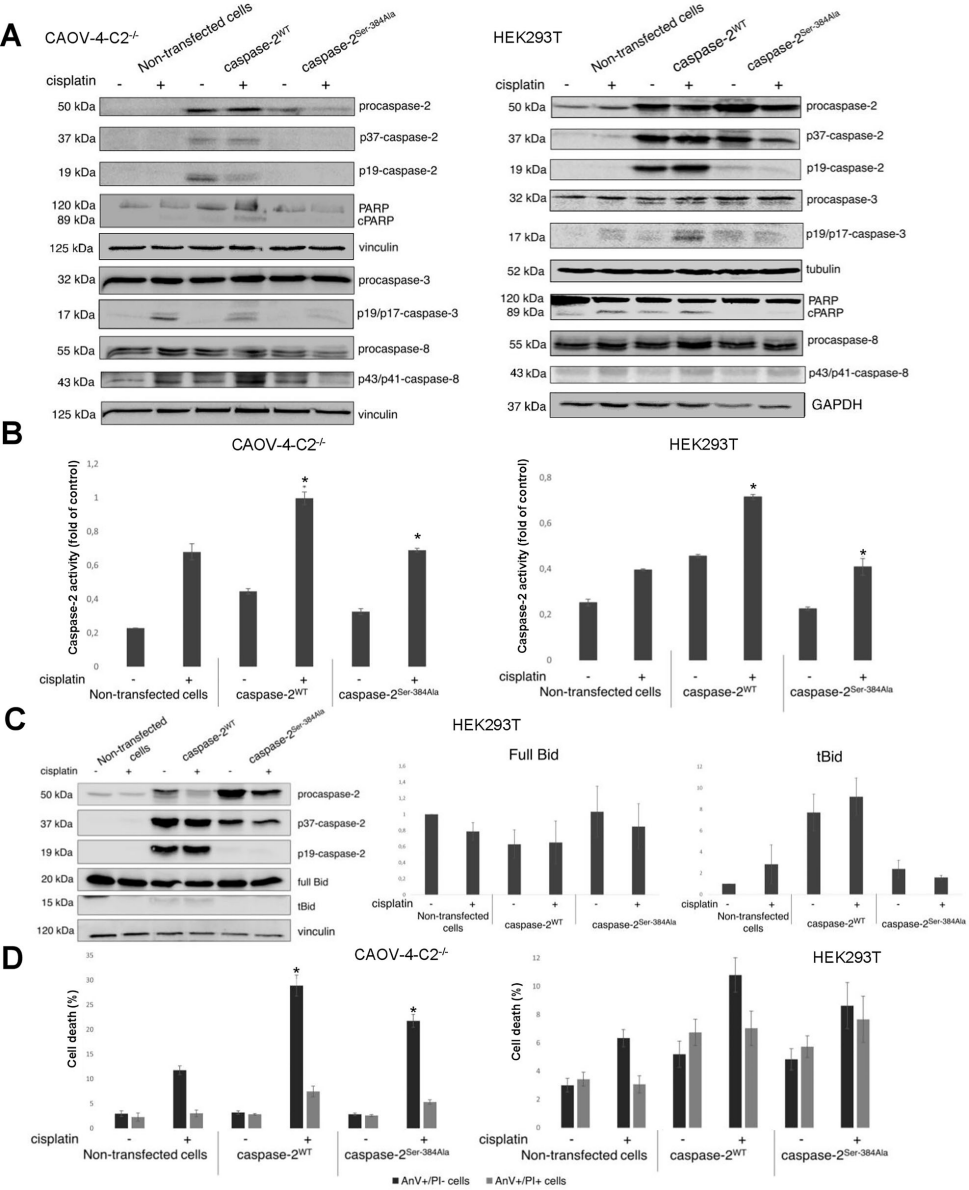


The original article has been corrected. We apologize to all readers for this mistake, which does not affect the conclusions of this article.

