# Supplementary material for: Requirement for Serine-384 in Caspase-2 processing and activity
Source: Cell Death Dis. 2020 Oct 3;11(10):825. doi: 10.1038/s41419-020-03023-6 (PMC7532978; doi:10.1038/s41419-020-03023-6)
Supplement: Supplementary file 2 — Supplementary Figure Legends [file 41419_2020_3023_MOESM2_ESM.docx]

**SUPPLEMENTARY MATERIALS**

**Movie.** The conformational change of Arg-378 in the Ser-384Ala mutant form of caspase-2. A part of the 10 ns MD trajectory corresponding to the time interval of 3.7–4.7 ns is shown.

**Figure S1:** The list of potential novel caspase-2 phosphorylation sites identified by bioinformatics algorithms. The phosphorylation events of underlined residues were shown experimentally ^12-14^.

**Figure S2:** The S380A/S307A/S220A substitutions do not decrease the caspase-2 peptidase activity. Caspase-2 activity was measured using a substrate - Ac-VDVAD-AMC in lysates of HEK293T cells that were non-transfected or transfected with caspase-2WT and caspase-2 S380A/S307A/S220A. Both cell lines were treated with 70 µM cisplatin for 18 h and caspase activity was measured. Results are presented as the mean of three distinct experiments ± SD in each time point.

**Figure S3:** The key role of caspase-2 in DNA damage-induced apoptosis. The processing of caspase-2 and caspase-3 and the appearance of PARP cleavage product p89 (cPARP) in wt CAOV-4 cells, in CAOV-4-shRNA-caspase-2 with downregulated level of capsase-2, in knockout caspase-2 cell line - CRISPR/Cas9 caspase-2^-/-^ CAOV-4 cells and in control CRISPR/Cas9 CAOV-4 cells. Cell lines were treated with 70 µM cisplatin for 18 h and caspase processing was analyzed by western blot. The bands corresponding to cleaved fragments of caspase-2, caspase-3, and PARP are marked as p37-caspase-2/p19-caspase-2, p19/17-caspase-3, and cPARP, correspondingly.

**Figure S4:** The amount of procaspase-2 and cleaved fragments of caspase-2, caspase-3 and PARP were quantified by densitometry analysis and normalized on loading control level. Results are presented as the mean of four distinct experiments ± SEM.

**Figure S5**: LC-MSMS analysis of Caspase-2 did not reveal the phosphorylation of Ser-157 and Ser-220. Annotated spectra are shown with all of the fragments that matched an expected fragment from *in silico* digestion (in color). Below each spectrum is a table with calculated fragment ion masses for each peptide; colored values match corresponding peaks in the spectrum. No phosphorylation was detected on Ser-157 (peptide LSTDTVEHSLDNK) and Ser-220 (peptide SGGDVDHSTLVTLFK)

**Figure S6:** Geometric characteristics of the planar carboxylate and tetrahedral phosphate groups (van der Waals representation).

**Legend to Expanded View**

Coordination of caspase-2 active center by Serine-384.
