# Supplementary figures and images for: Requirement for Serine-384 in Caspase-2 processing and activity
Source: Cell Death Dis. 2020 Oct 3;11(10):825. doi: 10.1038/s41419-020-03023-6 (PMC7532978; doi:10.1038/s41419-020-03023-6)

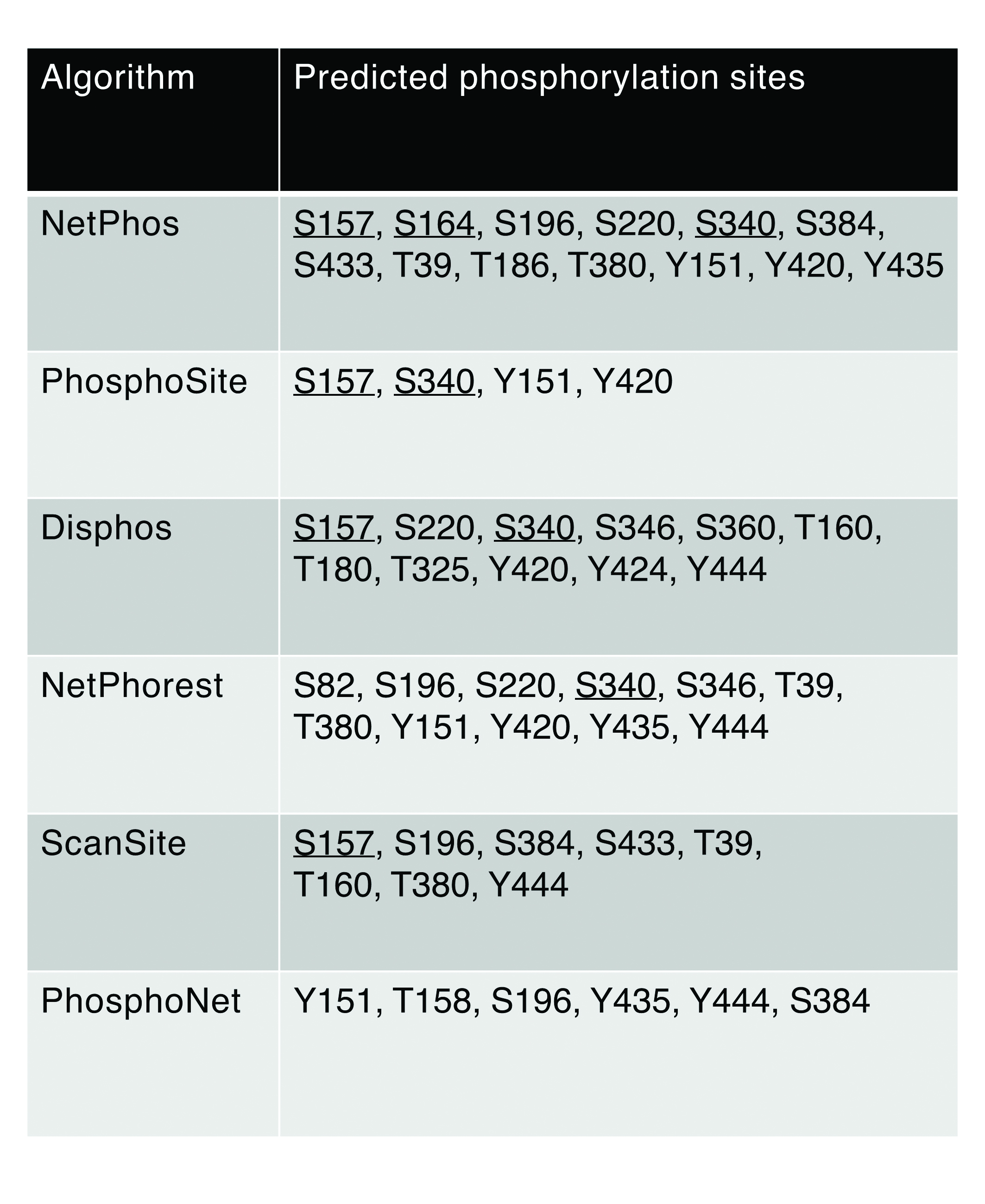

Supplement: Supplementary file 3 — Supplementary Figure 1 [file 41419_2020_3023_MOESM3_ESM.jpg]

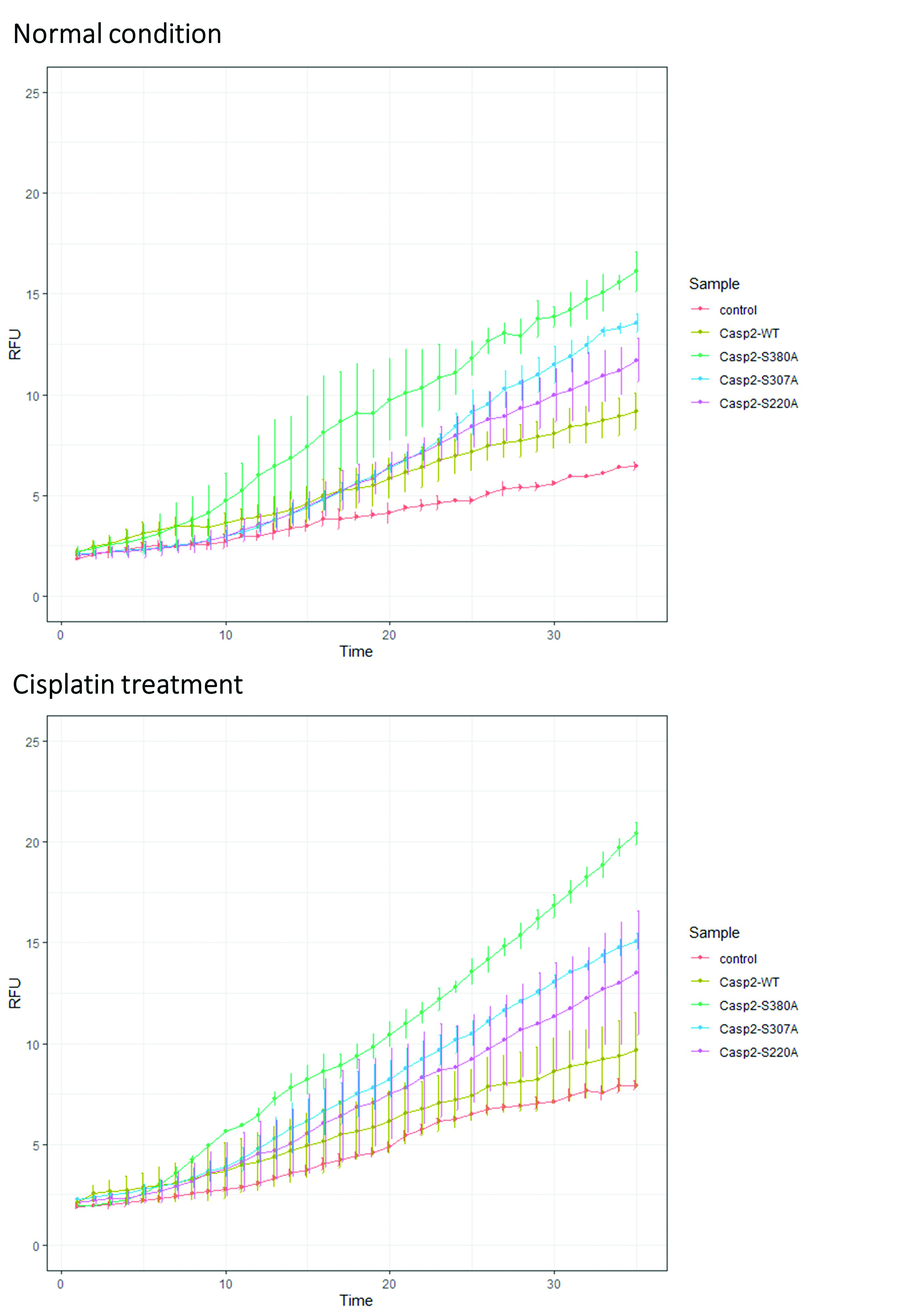

Supplement: Supplementary file 4 — Supplementary Figure 2 [file 41419_2020_3023_MOESM4_ESM.jpg]

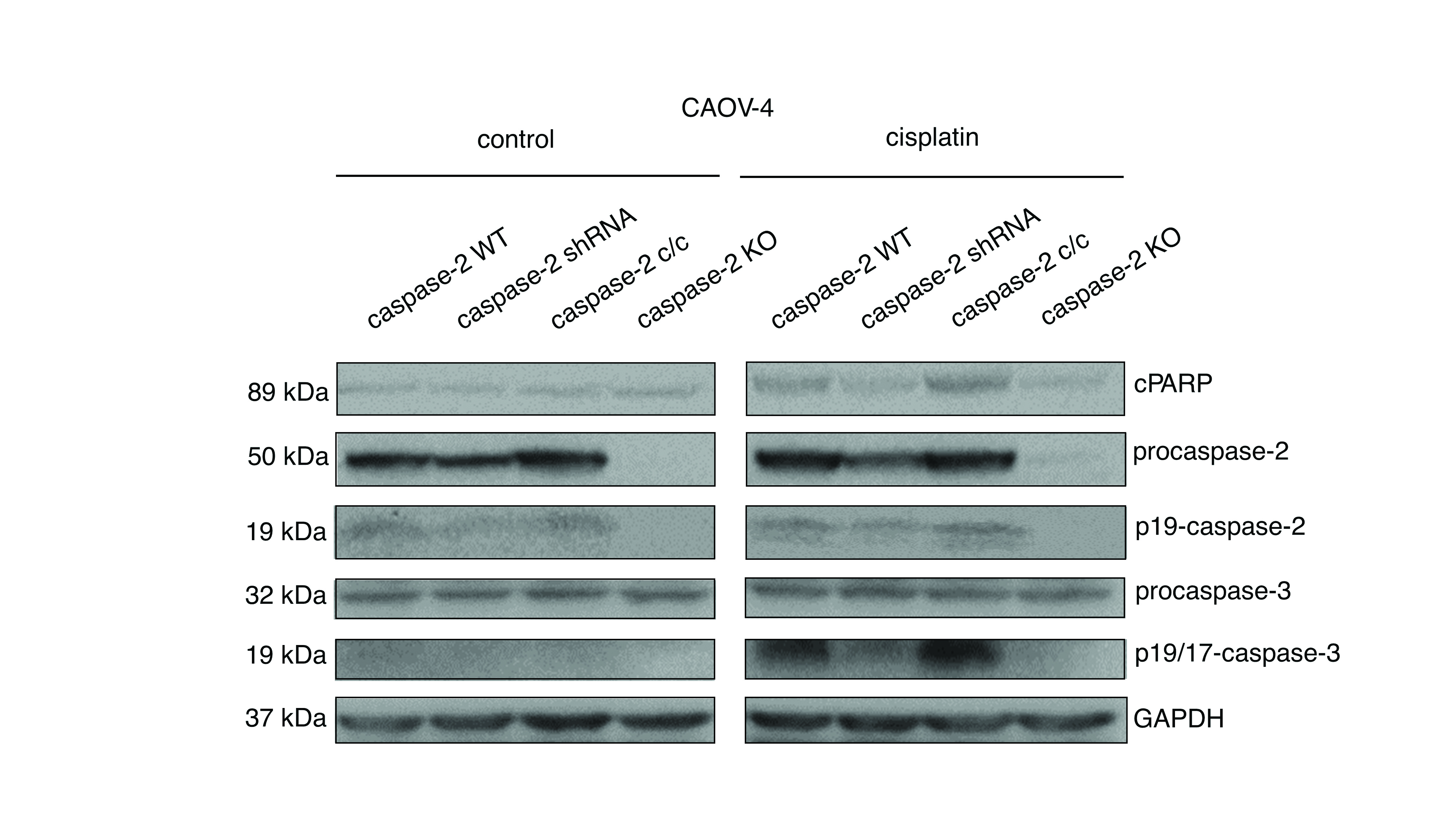

Supplement: Supplementary file 5 — Supplementary Figure 3 [file 41419_2020_3023_MOESM5_ESM.jpg]

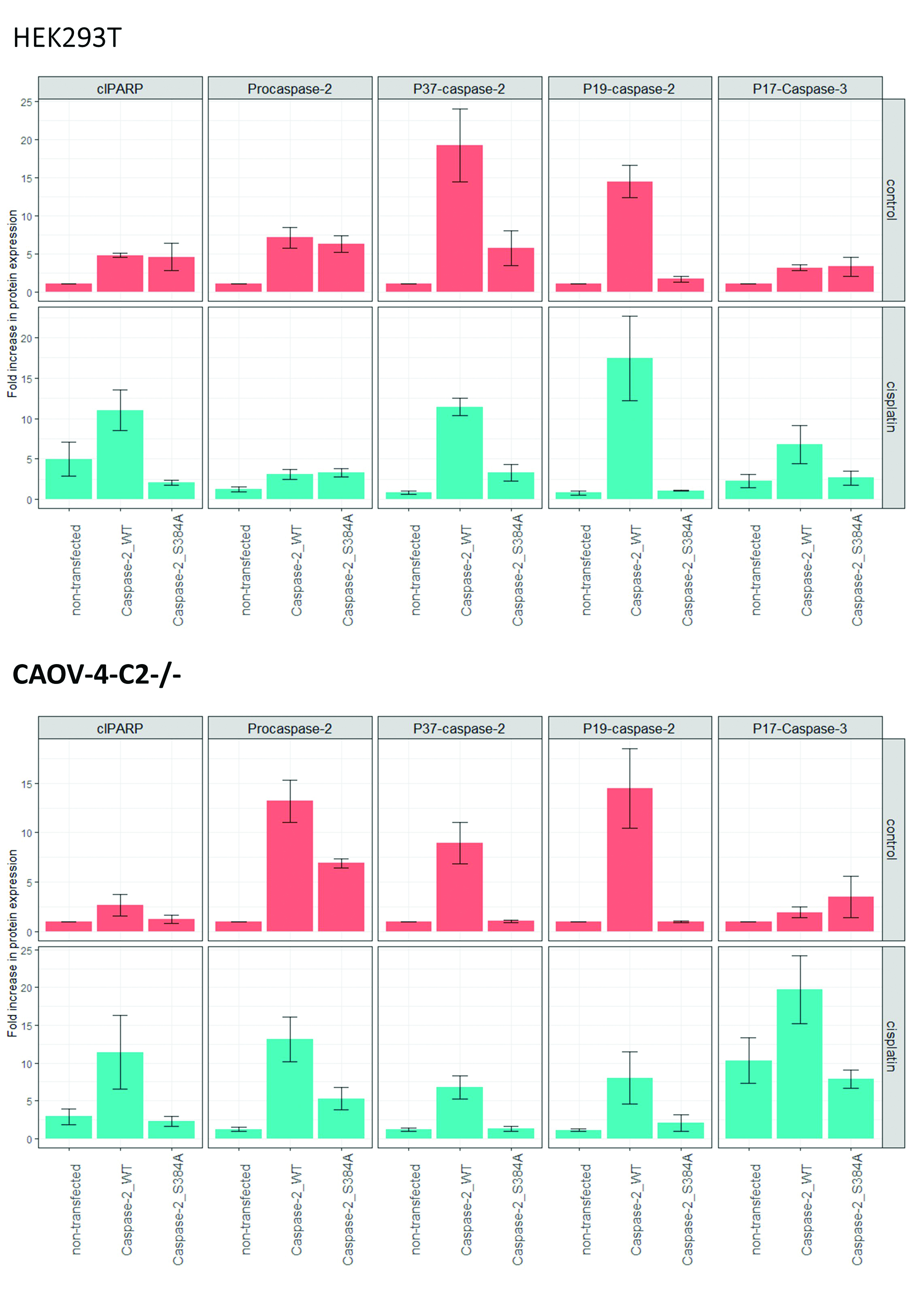

Supplement: Supplementary file 6 — Supplementary Figure 4 [file 41419_2020_3023_MOESM6_ESM.jpg]

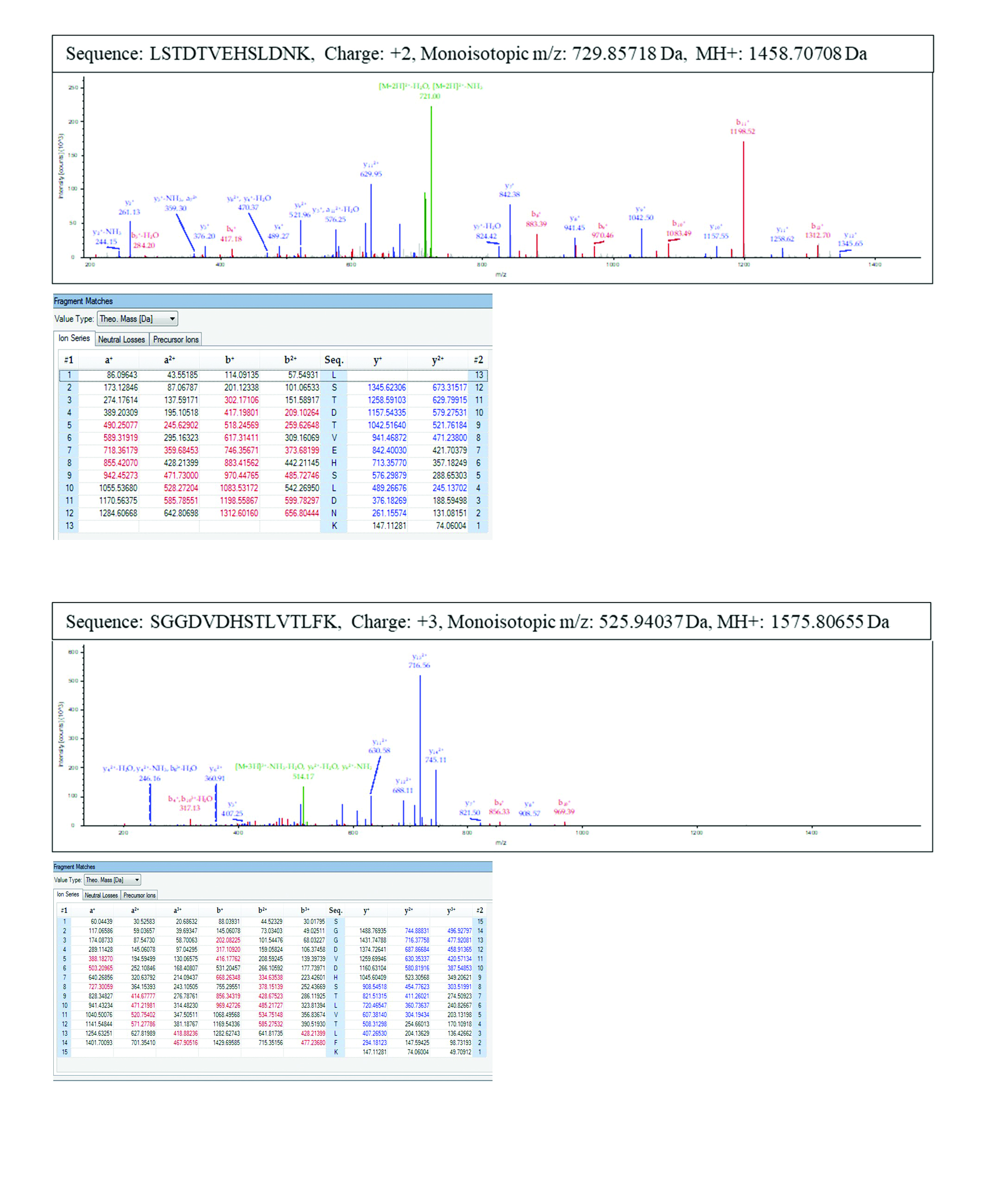

Supplement: Supplementary file 7 — Supplementary Figure 5 [file 41419_2020_3023_MOESM7_ESM.jpg]

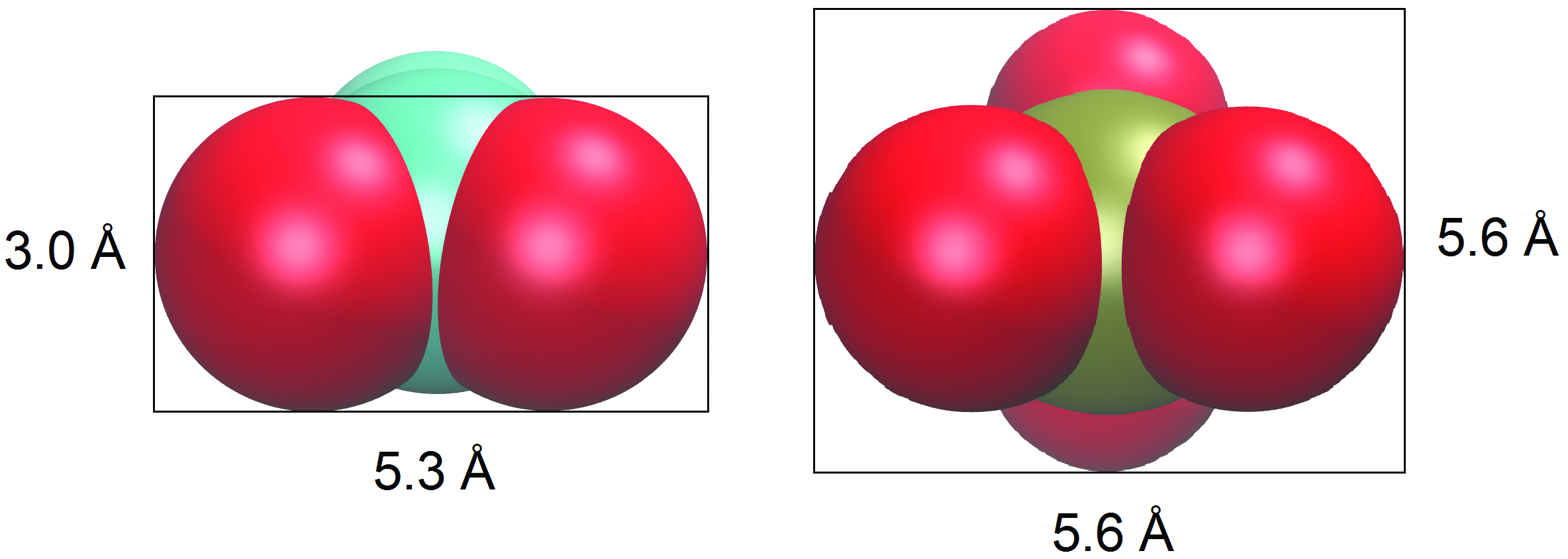

Supplement: Supplementary file 8 — Supplementary Figure 6 [file 41419_2020_3023_MOESM8_ESM.tif]
